# Effect of Acute Mental Stress on Heart Rate and QT Variability in Postmyocardial Infarction Patients

**DOI:** 10.5402/2012/912672

**Published:** 2012-07-15

**Authors:** Damiano Magrì, Gianfranco Piccirillo, Raffaele Quaglione, Annalaura Dell'Armi, Marilena Mitra, Stefania Velitti, Daniele Di Barba, Andrea Lizio, Damiana Maisto, Francesco Barillà

**Affiliations:** ^1^Dipartimento di Medicina Clinica e Molecolare, Azienda Ospedaliera S. Andrea, “Sapienza” Università degli Studi di Roma, 00185 Rome, Italy; ^2^Dipartimento di Scienze Cardiovascolari, Respiratorie, Nefrologiche e Geriatriche, Prima Clinica Medica, Policlinico Umberto I, “Sapienza” Università degli Studi di Roma, 00185 Rome, Italy

## Abstract

Emotionally charged events are associated with an increased risk of sudden cardiac death (SCD). In this study we assessed RR and QT variability index (QTVI) at baseline during anger recall test (AR). We calculated QTVI from a 5-min ECG recording and from a 10-beats segment around the presumed maximum sympathetic activation in thirty post-myocardial infarction patients under *β* -blocker therapy and 10 controls underwent. In all groups, the low-frequency component of RR and SBP increased during AR. In all recordings, the QTVI calculated on a 5-min ECG recording and the QTVI_10 beats_ were higher in patients than in controls (*P* < 0.05). The QTVI during AR remained unchanged from baseline within each group. Conversely, during AR, the QTVI_10 beats_ in controls diminished significantly (*P* < 0.05) from baseline whereas in patients remained unchanged. The inability to buffer an acute stress-induced increase in sympathetic activity could explain why events charged with acute stress are associated with an increased risk of ventricular arrhythmias in this setting of patients and support the role of cognitive behavior stress management strategies.

## 1. Introduction

Sudden, unexpected events with a high emotional content [1, 2], such as, earthquakes [3–6], terrorist attacks [6–9], sports matches [10, 11], sexual activities [12, 13], or episodes of anger [14, 15] can lead to malignant ventricular arrhythmias and hence to sudden cardiac death (SCD), mainly in patients with structural heart disease.  The body reacts to these stressful circumstances by increasing sympathetic nervous activity and reducing vagal control of heart rate and blood pressure [16], such imbalance known to be a trigger for malignant ventricular arrhythmias [1, 2, 17]. Several Authors suggest that stressful events, such as, those experimentally induced through an anger recall test (AR), might lead also to a significant worsening in myocardial repolarization dispersion, another phenomenon thought to be a potential striking of lethal arrhythmias [18–20]. Moreover, evidence about a possible relationship between autonomic nervous system (ANS) activity and myocardial repolarization dispersion comes from a recent experimental study showing that the QT variability index (QTVI), marker of temporal dispersion in myocardial repolarization [21–24], tends to increase in a manner directly proportional to the increased sympathetic nerve activity [25]. However, stress-induced events typically cause nonuniform, short-lasting changes in ANS activity and, most likely, in myocardial repolarization dispersion [26, 27]. Indeed it could be reasonable that the QTVI analysis from a shorter period, possibly coinciding with the greatest sympathetic outflow, might supply useful insights into pathophysiological mechanisms underlying ventricular arrhythmias subsequent to an acute stress [28]. 

We therefore designed this pathophysiological study on postmyocardial infarction patients to evaluate ANS activity, as assessed by HRV power spectral analysis and QTVI changes in response to an acute mental stress induced by an AR. To accomplish our hypothesis we obtained QTVI values not only from the classical 5 min ECG recording but also just from a 10-beat segment around the presumed maximum sympathetic activation, arbitrarily indentified as that corresponding to the systolic blood pressure (SBP) peak. 

## 2. Methods

### 2.1. Study Sample and Data Acquisition

We selected 30 postmyocardial infarction outpatients: 20 patients with moderately reduced left ventricular ejection fraction (LVEF ≤ 45% Group) and 10 patients with preserved LVEF (LVEF ≥ 50% Group). We also recruited 10 healthy subjects without history of ischemic cardiomyopathy, from persons presenting spontaneously to our outpatient clinic for preventive reasons (Control Group). Before entering the study, all subjects underwent a complete history, physical examination, routine laboratory investigations, surface 12-lead ECG, and Doppler echocardiography evaluation. Exclusion criteria for postmyocardial infarction patients were recent myocardial infarction (less than 6 months), complete left bundle branch block (LBBB), asthma, malignancy, primary valve disease, atrial fibrillation, frequent extrasystole (one extrasystole per minute was permitted), or other arrhythmias likely to interfere with assessments. None of the patients were receiving antiarrhythmic therapy, except for *β*-blockers. Exclusion criteria for controls were smoke habits, diabetes (fasting glucose plasma level  >  6.1 mmol/L), cholesterol plasma level  >  5.2 mmol/L, arrhythmias,  LBBB or other conduction abnormalities, ultrasound evidence of significant hemodynamic stenosis or echocardiographic evidence of LV wall-motion abnormalities, valvular disease, or LV hypertrophy.

All participants gave their informed consent to the procedures and the institutional review board of the Department of the Science of Ageing, University of Rome, “La Sapienza” approved the study. The study complied with the ethical rules for human experimentation stated in the Declaration of Helsinki. 

### 2.2. Study Protocol and Offline Data Analysis

After a 15-minute rest lying down, each subject underwent two, 5 min, single ECG lead, and continuous beat-to-beat SBP recordings (Finometer Pro, FMS BV, Amsterdam, The Netherlands): the first under a baseline condition  (B) and the second one during a 5 min speech about a recent anger-provoking incident (AR). The analogical signals were acquired simultaneously and digitally converted with a custom-designed card (Keithley Metrabyte–DAS 1200 Series, Keithley Instruments GmbH, Munich, Germany) at a sampling frequency of 500 Hz per channel with 12-bit precision. All digitalized ECG recordings were analyzed by a single physician (G.P.) blinded to subjects circumstances. 

**Figure 1:**
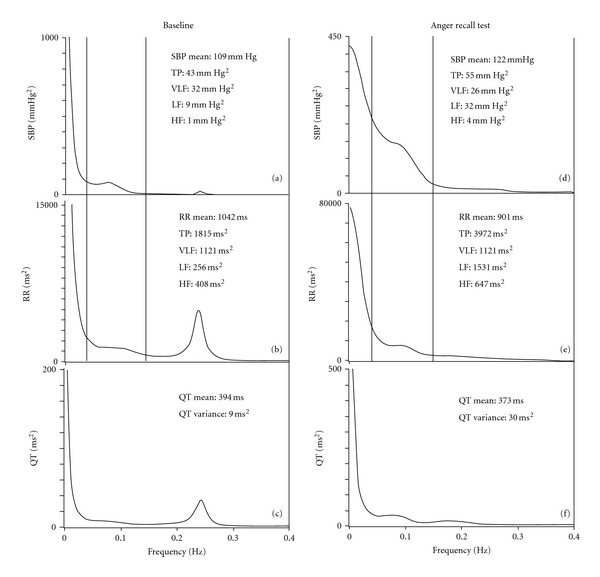
Example of short-period power spectra for systolic blood pressure (SBP), RR and QT interval recorded at baseline (a, b, c) and during the anger recall test (AR) (d, e, f). Note that SBP, RR, LF_SBP_, and TP_RR_ (total RR interval variance), LF_RR_, and QT variance all increase markedly during the AR.

The two 5 min segments obtained were then used to determine power spectral analysis with an autoregressive algorithm of RR and SPB variability [29–32]. We therefore determined the following spectral components: the total power of RR intervals and SBP (TP_RR_, TP_SBP_), a high-frequency (HF_RR_, HF_SBP_: from 0.15 to 0.40 Hz Eq), a low-frequency (LF_RR_, LF_SBP_: from 0.04 to 0.15 Hz Eq), and a very-low frequency component (VLF_RR_, VLF_SBP_: below 0.04 Hz Eq) (Figure 1). 

We also measured LF and HF central frequencies. Finally, we calculated a time-domain index: the standard deviations of successive RR interval differences (SDNN).

From the same two 5 min segments we calculated QT and RR mean (QT_m_ and RR_m_) and variances (QT_v_ and RR_v_). To make the end of the T wave easier to identify we used a software program based upon the algorithm for quantification of beat-to-beat fluctuations in QT interval variability proposed by Berger et al. [21].The QTVI was then obtained with the following classic formula:

(1) QTVI=log⁡10⁡{[(QTv)/(QTm)2][(RRv)/(RRm)2]}.

Finally the QTVI was also calculated, at baseline and during AR, from the 10-beat ECG segment (QTVI_10  beats_) around the SBP maximum value. Particularly, to select this short segment, we evaluated the maximum SBP value in baseline and during AR recordings and we calculated RR and QT intervals between 4 beats preceding and 6 beats following the SBP peak (a total of 10 beats). 

To validate the stability of repeated measures of QTVI_10  beats_ during baseline, similar recordings were repeated four days later in the same baseline conditions.

Software for data acquisition, storage, and for spectral analysis was designed and produced by our research group and is described in detail elsewhere [31–34]. 

Last, the corrected QT interval was obtained from the initial 12-lead ECG recording according to Bazett's (QT/RR^0.5^).

### 2.3. Statistical Analysis

Unless otherwise indicated all data are expressed as means ± SD. Data with skewed distribution (as evaluated by Kolgomorov-Smirnov test) are given as median and interquartile range [75th percentile–25th percentile]. Categorical variables were analyzed with the *χ* ^2^ test. One-way ANOVA and Bonferroni test were used to compare data for the normally distributed variables (including nonspectral data, RR_m_, QT_m_, and spectral coherence). Kruskal-Wallis and Mann-Whitney test were used to compare nonnormally distributed variables (including spectral data, QT_v_, RR_v_, QTVI). Paired *t*-test was used to compare data for the normally distributed variables between baseline and AR, whereas Wilcoxon test was used to compare nonnormally distributed variables. We also tested for the presence of a systematic change between successive measurements by the Wilcoxon matched paired test and verified whether the repeatability was dependent on the magnitude of the QTVI_10  beats_ by analysing the regression between standard deviation of two QTVI_10  beats_ measurement and the corresponding average in two different day sessions.

All data were evaluated with the database SPSS-PC+ (SPSS-PC+ Inc, Chicago, IL, USA). A *P* values of less than or equal to 0.05 were considered statistically significant.

## 3. Results

Neither age, BMI, SBP, plasma potassium nor sex distribution differed significantly between the three study groups, whereas heart rate, LVEF, QT_c_ differed significantly (Table 1). All postmyocardial infarction patients (n: 30), both in LVEF ≤ 45% and ≥50% Group, were receiving an optimized treatment with *β*-blockers (19 bisoprolol and 11 carvedilol). 

### 3.1. ECG and SBP Data from 5-Minute Epochs

In all study groups, the RR interval measured at baseline shortened significantly during AR and SBP increased (*P *< 0.05) (Table 2). 

In all study groups, power spectral analysis of RR and SBP variability showed that LF measured at baseline increased significantly during AR (*P* < 0.05). Most of the RR and SBP power spectral values, measured at baseline and during AR, were significantly lower in LVEF ≤ 45% than in LVEF ≥ 50% and in Control Group. During AR, the LVEF ≥ 50% Group showed LF and HF values significantly lower than those of the Control Group (*P* < 0.05), whereas, at baseline, only HF_RR_ values were significantly lower than those of the Control Group (*P *< 0.05). LF and HF central frequencies and HF_SBP_ showed no significant differences between groups (Table 2).

**Figure 2:**
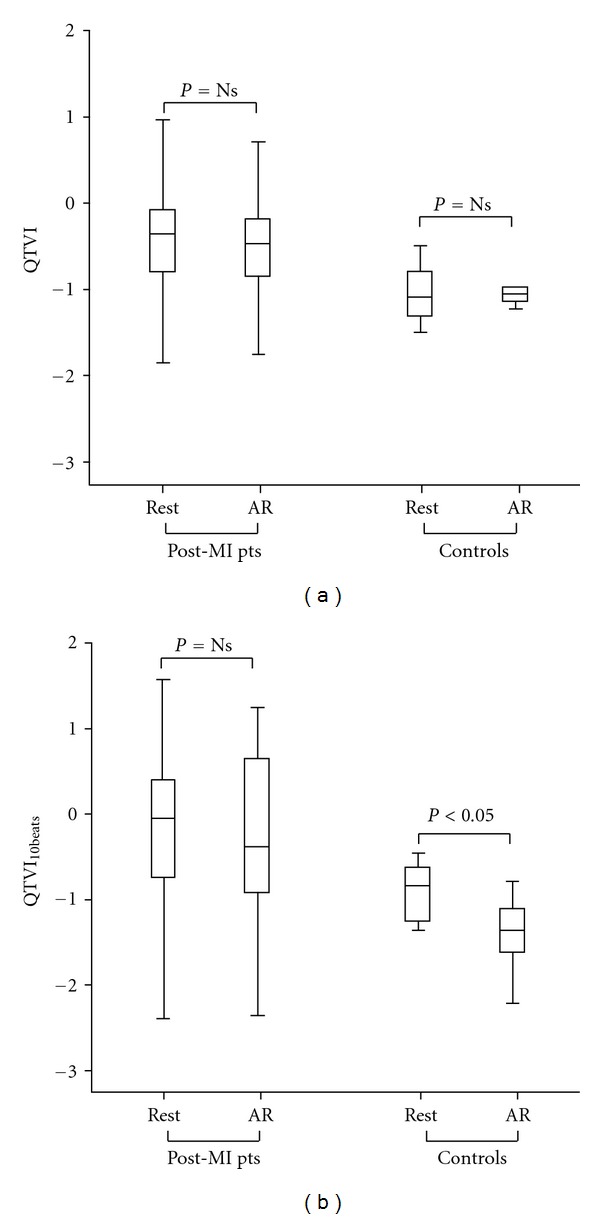
Comparison between QT variability index values at baseline (Rest) and during anger recall test (AR) in the whole postmyocardial infarction patients (post-MI pts) and healthy control subjects (Controls). (a) QT variability index values obtained with the classical method on 5 min continuous ECG epochs (QTVI). (b) QT variability index values obtained from a 10-beat segment around the presumed maximum sympathetic activation (QTVI_10  beats_). In the box plots, the central line represents the median distribution. Each box spans from 25th to 75th percentile points, and error bars extend from 10th to 90th percentile points.

At baseline and during AR, QTVI values were significantly higher in the LVEF ≤ 45% Group than in the other two study groups (*P* < 0.05). Eventually, only during AR, the LVEF ≥ 50% Group showed a significantly higher QTVI values than those of the Control Group (*P* < 0.05). QTVI values did not significantly change within group during AR with respect to baseline condition (Table 2, Figure 2).

### 3.2. ECG and SBP Data from 10  beats Epochs

Under both experimental conditions (baseline and AR), mean SBP_10  beats_ and RR_10  beats_ did not significantly differed between study groups whereas, during AR, mean RR_10  beats_ measured at baseline decreased significantly, whereas mean SBP_10  beats_ increased significantly in all study groups (*P* < 0.05) (Table 3).

At baseline and during AR, RR_10  beats_ variance was significantly lower in both postmyocardial infarction groups than in controls, whereas no difference was found between groups for QT_10  beats_ mean and QT_10  beats_ variance (Table 3). During AR, RR_10  beats_ variance increased (*P* < 0.05), whereas QT_10  beats_ mean decreased significantly (*P* < 0.05) only in the Control Group (Table 3).

QTVI_10  beats_ was significantly lower in controls than postmyocardial infarction patients groups at baseline and during AR (*P* < 0.05) (Table 3). At baseline, QTVI_10  beats_ was significantly lower in LVEF ≥ 50% than in LVEF ≤ 45% Group (*P* < 0.05) (Table 3). During AR, both postmyocardial infarction groups showed a significantly higher QTVI_10  beats_ than controls. QTVI_10  beats_ decreased significantly from baseline to AR condition (*P* < 0.05) only in control group (Table 3, Figure 2).

Finally we did not find any significant differences in QTVI_10  beats_ between baseline recordings obtained at the beginning and four days after the end of the study in our sample (−0.44[1.20] versus −0.48[1.11], *P* = 0.347). The standard deviation was dependent on QTVI_10  beats_ magnitude (*R* ^2^ = 0.11, *P* = 0.037), indicating that the repeatability of QTVI_10  beats_ tends to decrease as QTVI_10  beats_ decreases.

## 4. Discussion

This pathophysiological study offers interesting insights into mechanisms underlying ventricular arrhythmias triggering during an acute mental stress in postmyocardial infarction patients. Furthermore our findings seem to suggest the QTVI_10  beats_, rather than the QTVI obtained from a whole 5-min ECG recording, as a possible mirror of these mechanisms. 

### 4.1. ANS Control during Acute Mental Stress

As expected, the significant increase in LF_RR_ power during AR differed in magnitude between patients and controls: LF_RR_ values in controls were almost 4 times higher than those in postmyocardial infarction patients at baseline and 5 times higher during AR. Because studies in humans and experimental animal research in recent years have clarified that under normal conditions an increased LF_RR_ power is associated with increased sympathetic activity and reduced vagal sinus activity [29–32, 35], this cardiovascular variable is considered a reasonably reliable marker of sympathetic activation. Conversely, during heart failure, even though reduced LF_RR_ values are associated with an increased risk of SCD [36–38], LF_RR_ seems unreliable for assessing ANS control [35]. Interestingly, in the actual study setting, it seems to be a residual ANS reactivity to acute mental stress in postmyocardial infarction patients, thus accounting for a key role of acute ANS changes as a trigger of malignant ventricular arrhythmias. Indeed AR led to an mild but significant increase of LF_RR_ and heart rate to a similar extent in postmyocardial infarction patients and in control subjects. A possible explanation is that in patients we studied, the concomitant *β*-blocker therapy attenuated the effects of high catecholamine levels on *β*-adrenergic receptors thus helping to maintain sinus node responsiveness to AR-mediated sympathetic stimulation. A previous study, reporting an increase in LF_RR_ in CHF patients treated with carvedilol, argues in favour of this hypothesis [39].

### 4.2. QTVI during Acute Mental Stress

Our finding that the QTVI from a 5 min ECG recording remained almost unchanged during the AR test in postmyocardial infarction patients discords slightly with recent experimental data obtained in dogs with pacing-induced heart failure [25]. In fact in the previous study, as in the actual, QTVI values under healthy conditions remained statistically unchanged as sympathetic activity increased from low to high levels. Conversely QTVI values worsened after heart failure induction while, in the actual study setting, the presumed sympathetic burst failed to increase this index. It is likely that *β*-blocker therapy in postmyocardial patients strongly attenuated the AR-mediated increase in myocardial repolarization dispersion, as we previously reported [40]. Our hypothesis receives further support from another previous study conducted on chronic heart failure patients on *β*-blocker therapy, showing that the QTVI from the whole 5 min ECG segment remained statistically unchanged in response to other sympathetic stimulation modalities, such as, tilt [41]. Nevertheless it needs to be underlined that current study does not question about the QTVI ability in stratifying SCD risk in heart failure patients under stable *β*-blocker therapy [23, 24]. Indeed it is of note that also postinfarction patients evaluated in current study showed QTVI values significantly higher than those of healthy controls, thus accounting for their greater arrhythmic risk. 

### 4.3. QTVI_10  beats_ during Acute Mental Stress

The most interesting and completely novel finding in this study was that during the presumed maximum sympathetic outflow in all postmyocardial infarction patients the QTVI_10  beats_ remains statistically unchanged, whereas it significantly decreases in healthy subjects. This datum raises an intriguing hypothesis about a possible different QTVI_10  beats_ ability in exploring sudden changes in ANS activity and/or temporal myocardial repolarization dispersion due to acute stress with respect to the classic QTVI. In fact, in accordance with the general formula (see Section 2), QTVI values increase, and hence worsen, in a measure proportional to the reduced RR-interval variance and duration and/or to the increased QT interval variance and duration [21]. Interestingly in control group RR_10  beats_ variance increased almost 7 times from baseline during AR, whereas in postmyocardial infarction group it remained almost unchanged. Hence precisely for this reason the QTVI_10  beats_ decreased significantly only in control group. From a pathophysiological viewpoint, the more prominent increase in RR_10  beats_ variance in controls during AR could reflect an increased baroreflex activity, a cardiovascular response typically impaired after myocardial infarction. Under this viewpoint we might interpret the reduction in the QTVI_10  beats_ as an indirect marker of a vagal protective mechanism, present in healthy persons but lost in patients after myocardial infarction. 

Another, somewhat, unexpected finding was the similar QTVI_10  beats_ behavior found in postmyocardial infarction patients regardless the LVEF values. This datum support a widespread belief that the the arrhythmic risk is not exhaustively predicted by the reduced LVEF alone.

### 4.4. Acute Stress and Arrhythmic Risk

Several epidemiological studies showed that psychological stress, that is, emotionally devastating disasters, such as, earthquake or war, lead to an increase in potentially fatal arrhythmias events [1–7]. Moreover this relationship has been extensively explored in recent studies involving patients with implantable cardioverter-defibrillators [8, 9]. The actual study supplies further evidence in understanding mechanisms linking anger and arrhythmias in postmyocardial infarction patients, supporting a key-role of the ANS imbalance with a rapid sympathetic outflow and/or a vagal tone withdrawal, as well of an acute change in electrophysiological myocardial properties. Because of its equation (see formula in the methods), the QTVI_10  beats_ exploits the characteristics of both heart rate and QT variability, thus seeming a suitable index for a short-lasting ANS and myocardial transmural dispersion changes in response to an acute stress [27, 28]. Indeed, albeit is not proved in current study, another pathophysiological mechanisms, besides the RR_10  beats_ variance increase, might argue in favour of a potential advantage in using QTVI_10  beats_, that is, the influence of the remodeling of the slow component of the delayed rectifier potassium current (IKS). It could be hypothesized that the significant downregulation of IKS, just reported in heart failure patients, could be responsible of a delay in action potential duration shortening during the initial few seconds of sympathetic stimulation [42, 43]. Therefore, the spatiotemporal heterogeneity of myocardial repolarization would be more apparent for a short period during AR, whereas it will gradually reduce as time lag increases, allowing the QTVI_10  beats_ to better explore this phenomenon. Unfortunately, perhaps because of the concomitant optimized *β*-blocker therapy, we did not find any significant increase in QT variance within the 10-beat ECG segments around the SBP peak. Nevertheless the influence of IKS remodeling on QTVI_10  beats_ remains an intriguing, albeit purely speculative, hypothesis that needs further investigation. 

Finally the actual study strengthens all approaches aimed to decrease the detrimental effects of anger and other negative emotions on arrhythmogenesis. Indeed it has just been showed in pilot studies that specific behavioral interventions significantly improve clinical management of patients with implantable cardioverter defibrillators, also by decreasing the arrhythmias rate [43–45]. In this complex scenario, the QTVI_10  beats_ obtained during AR might facilitate the identification of patients with higher SCD risk that, despite an optimized medical therapy, should receive a dedicated biopsychosocial approach.

## 5. Study Limitations

The small study sample, together with the present lack of prospective data on cardiac events, represents an obvious limitation that allows us just to hypothesize about each of abovementioned pathophysiological mechanisms. However, we believe that our preliminary findings deserves attention, mainly because of the importance in seeking better insights into these mechanisms. Moreover, QTVI_10 beats_ seems to be a sufficiently reliable and reproducible test since that all the three study groups showed similar results in two successive baseline recordings and the positive relationship between mean and standard deviation indicated that this test tended to lose his sensitivity mainly for very negative values (i.e., normal subjects).

We clearly recognize the potential weakness of SBP peak as a marker of maximum sympathetic activation. Nevertheless, in the absence of direct ANS activity measures, we decided to use the SBP instead of the heart rate peak for three specific reasons. First patients were under *β*-blocker treatment and, being nonethical to discontinue this therapy, heart rate might be misleading as a sympathetic marker. Second, we hypothesized the arteriolar resistances, underlying the SBP peak, as a more effective marker of sympathetic activity in comparison with heart rate peak, because postmyocardial patients often show a sinus dysfunction. Third, heart rate is influenced both by sympathetic and vagal activity, whereas SBP is predominantly sympathetic mediated.

Finally we cannot exclude that the observed difference between postmyocardial infarction patients and healthy controls could be partially due to the concomitant *β*-blockers therapy in the first group. However, we wish to underline that *β*-blockers therapy is known to be associated with a heart rate and QT variability improvement in these patients, thus it is likely that our results might be weakened rather than strengthened from *β*-blockers therapy in postmyocardial infarction groups. Moreover as well we considered it nonethical to administer *β*-blockers to healthy subjects, the same consideration should be done about a possible study protocol providing *β*-blockers discontinuation in patients at moderate-to-high risk of SCD.

## 6. Conclusions

Our finding observed, despite a concomitant stable *β*-blocker therapy, supports the undeniable link between ANS activity, myocardial repolarization, and arrhythmic risk. The impairedability to buffer acute sympathetic activity, evidenced by the absent reduction in the QTVI_10  beats_ during AR, could partially explain why events engendering acute mental stress are associated with an increased risk for SCD from malignant ventricular arrhythmias in postmyocardial infarction patients. The actual study, albeit indirectly, argues in favour of behavioral approaches in this patients where it should be essential to couple an optimized medical therapy with a specific cognitive behavior stress management course aimed to minimize the detrimental effects of anger and other negative emotions on arrhythmogenesis.

## Figures and Tables

**Table 1:** General characteristics in the three study groups.

Variables	LVEF ≤ 45% group N: 20	LVEF ≥ 50% group N: 10	Control group N: 10	*P*
Age, years	49 ± 11	52 ± 5	49 ± 9	Ns
Male/Female	16/4	8/2	6/4	Ns
BMI, kg/m^2^	27.2 ± 4.3	26.8 ± 1.0	24.7 ± 2.6	Ns
HR, beats/min	69 ± 9	67 ± 7	61 ± 7	Ns
QT_Bazett_	0.417 ± 0.050	0.384 ± 0.031	0.378 ± 0.021	Ns
LVEF, %	33 ± 7^†§^	53 ± 4^∗§^	65 ± 6^†∗^	<.001
NYHA class, I/II/III	0/9/11^†§^	6/4/0^∗§^	10/0/0^†∗^	<.001
Potassium, mEq/dL	4.19 ± 0.21	4.19 ± 0.17	4.24 ± 0.27	Ns

Data are expressed as mean ± SD. BMI: body mass index; HR: heart rate; LVEF: left ventricular ejection fraction; NYHA: New York Heart Association; *P* < 0.05 LVEF < 45% Group versus Controls; ^§^ *P* < 0.05 LVEF < 45% Group versus LVEF > 50% Group; ^∗^ *P* < 0.05 LVEF > 50% Group versus Controls.

**Table 2:** RR and SPB power spectral data and QTVI values obtained from the analysis of the whole 5 min ECG segment in the three study groups.

Variables	LVEF ≤ 45% group N: 20	LVEF ≥ 50% group N: 10	Control group N: 10	*P* ANOVA	LVEF ≤ 45% group N: 20	LVEF ≥ 50% group N: 10	Control group N: 10	*P* ANOVA
	Baseline				During Anger Recall test		
RR mean, ms	877 ± 145^‡^	873 ± 86^‡^	1006 ± 110^‡^	Ns	848 ± 149	833 ± 97	908 ± 86	Ns
SBP mean, mmHg	108 ± 19^‡^	122 ± 32^‡^	113 ± 25^‡^	Ns	119 ± 24	132 ± 34	124 ± 28	Ns
SDNN, ms	23 ± 10^††^	31 ± 12	42 ± 12	<.001	28 ± 14^††^	37 ± 12	51 ± 14	<.001
TP_RR_, ms^2^	481 [542]^‡††§^	977 [799]^‡^	1814 [1352]	<.001	533 [1183]^††§^	1456 [1509]	2121 [1628]	<.001
VLF_RR_, ms^2^	293 [303]^††§^	527 [714]	1091 [568]	<.001	209 [339]^†§^	967 [822]	1030 [3741]	.004
LF_RR_, ms^2^	71 [127]^‡†§^	233 [315]^‡^	308 [367]^‡^	.003	183 [286]^††§^	411 [755]^∗^	918 [625]	<.001
HF_RR_, ms^2^	52 [101]^†^	51 [127]^∗^	173 [472]^†^	.006	58 [144]^†^	94 [93]^∗^	236 [553]	.006
TP_SPB_, mmHg^2^	14 [9]^‡†§^	26 [21]^‡^	38 [37]^†^	.003	33 [21]^§^	65 [112]	49 [106]	.027
VLF_SPB_, mmHg^2^	7 [5]^††§^	19 [22]^‡^	30 [33]	<.001	13 [18]^§^	48 [9]^§^	21 [73]	.039
LF_SPB_, mmHg^2^	2 [3]^‡†^	4 [3]^‡^	6 [9]^‡^	.003	7 [4]^†§^	13 [9]^§^	15 [12]	.002
HF_SPB_, mmHg^2^	2 [3]^‡^	2 [1]^‡^	1 [2]	Ns	4 [5]	4 [5]	2 [4]	Ns
LF CF, Hz	0.09 ± 0.03	0.08 ± 0.16	0.10 ± 0.02	Ns	0.09 ± 0.03	0.08 ± 0.02	0.09 ± 0.04	Ns
HF CF, Hz	0.25 ± 0.02	0.24 ± 0.00	0.25 ± 0.00	Ns	0.22 ± 0.04	0.22 ± 0.03	0.21 ± 0.04	Ns
QTVI	−0.25 [0.67]^†§^	−0.83 [0.70]	−1.10 [0.55]	.001	−0.37 [0.82]^†§^	−0.76 [0.44]^∗^	−1.05 [0.23]	<.001

Values are expressed as mean ± SD or median [interquartile range 75th percentile–25th percentile].

TP: total spectral power; VLF: very-low frequency; LF: low frequency; HF: high frequency; CF: central frequency; SBP: systolic blood pressure.

^‡^ *P* < 0.05 data at baseline versus data after Anger Recall test; ^††^ *P* < 0.001 LVEF ≤ 45% Group versus Controls; ^†^ *P* < 0.05 LVEF ≤ 45% Group versus Controls;

^§^ *P* < 0.05 LVEF ≤ 45% Group versus LVEF ≥ 50% Group; ^∗^ *P* < 0.05 LVEF ≥ 50% Group versus Controls.

**Table 3:** Nonspectral RR, SPB, and QT data obtained from the analysis of the 10-beat segment collected during SBP maximum peak in the three study groups.

Variables	LVEF ≤ 45% group N: 20	LVEF ≥ 50% group N: 10	Control group N: 10	*P*	LVEF ≤ 45% group N: 20	LVEF ≥ 50% group N: 10	Control group N: 10	*P *
	Baseline				During Anger Recall test		
SBP_10 beats_, mmHg	113 ± 20^‡^	133 ± 30^‡^	127 ± 25^‡^	Ns	131 ± 25	145 ± 31	139 ± 27	Ns
RR_10 beats_ mean, ms	877 ± 143^‡^	855 ± 93^‡^	1013 ± 110^‡^	Ns	824 ± 139	808 ± 76	886 ± 102	Ns
RR_10 beats_ variance, ms^2^	312 [462]^†^	205 [827]^∗^	538 [3385]^‡^	.032	307 [1038]^†^	385 [1930]^∗^	3426 [3781]	.004
QT_10 beats_ mean, ms	372 ± 49	351 ± 46	382 ± 30^‡^	Ns	366 ± 47	340 ± 39	364 ± 31	Ns
QT_10 beats_ variance, ms^2^	27 [54]	18 [26]	11 [18]	Ns	45 [75]	27 [28]	18 [20]	Ns
QTVI_10 beats_	0.01 [0.97]^†§^	−0.77 [2.07]	−0.84 [0.63]^‡^	.003	−0.41 [1.80]^†^	−0.50 [1.45]^∗^	−1.35 [0.60]	.003

Values are expressed as mean ± SD or median [interquartile range 75th percentile–25th percentile]. Abbreviations as in Tables 1 and 2.

^‡^ *P* < 0.05 data at baseline versus data after Anger Recall Test; ^†^ *P* < 0.05 LVEF ≤ 45% Group versus Controls; ^§^ *P* < 0.05 LVEF ≤ 45% Group versus LVEF ≥ 50% Group; ^∗^ *P* < 0.05 LVEF ≥ 50% Group versus Controls.

## References

[1] Ziegelstein RC (2007). Acute emotional stress and cardiac arrhythmias. *Journal of the American Medical Association*.

[2] Dimsdale JE (2008). Psychological stress and cardiovascular disease. *Journal of the American College of Cardiology*.

[3] Leor J, Poole WK, Kloner RA (1996). Sudden cardiac death triggered by an earthquake. *The New England Journal of Medicine*.

[4] Kloner RA, Leor J, Poole WK, Perritt R (1997). Population-based analysis of the effect of the Northridge Earthquake on cardiac death in Los Angeles County, California. *Journal of the American College of Cardiology*.

[5] Kario K, Ohashi T (1997). Increased coronary heart disease mortality after the Hanshin-Awaji earthquake among the older community on Awaji Island. *Journal of the American Geriatrics Society*.

[6] Chi JS, Poole WK, Kandefer SC, Kloner RA (2003). Cardiovascular mortality in New York City after September 11, 2001. *American Journal of Cardiology*.

[7] Chi JS, Speakman MT, Poole WK, Kandefer SC, Kloner RA (2003). Hospital admissions for cardiac events in New York City after September 11, 2001. *American Journal of Cardiology*.

[8] Steinberg JS, Arshad A, Kowalski M (2004). An Increased incidence of life-threatening ventricular arrhythmias in implantable defibrillator patients after the World Trade Center attack. *Journal of the American College of Cardiology*.

[9] Shedd OL, Sears SF, Harvill JL (2004). The World Trade Center attack: increased frequency of defibrillator shocks for ventricular arrhythmias in patients living remotely from New York City. *Journal of the American College of Cardiology*.

[10] Carroll D, Ebrahim S, Tilling K, Macleod J, Davey Smith G (2002). Admissions for myocardial infarction and World Cup football: database survey. *British Medical Journal*.

[11] Wilbert-Lampen U, Leistner D, Greven S (2008). Cardiovascular events during World Cup Soccer. *The New England Journal of Medicine*.

[12] Muller JE, Mittleman MA, Maclure M, Sherwood JB, Toller GH (1996). Triggering myocardial infarction by sexual activity: low absolute risk and prevention by regular physical exertion. *Journal of the American Medical Association*.

[13] Möller J, Ahlbom A, Hulting J (2001). Sexual activity as a trigger of myocardial infarction. A case-crossover analysis in the Stockholm Heart Epidemiology Programme (SHEEP). *Heart*.

[14] Mittleman MA, Maclure M, Sherwood JB (1995). Triggering of acute myocardial infarction onset by episodes of anger. *Circulation*.

[15] Möller J, Hallqvist J, Diderichsen F, Theorell T, Reuterwall C, Ahlbom A (1999). Do episodes of anger trigger myocardial infarction? A case-crossover analysis in the Stockholm Heart Epidemiology Program (SHEEP). *Psychosomatic Medicine*.

[16] Appels CWY, Bolk JH (2009). Sudden death after emotional stress: a case history and literature review. *European Journal of Internal Medicine*.

[17] Willich SN, Maclure M, Mittleman M, Arntz HR, Muller JE (1993). Sudden cardiac death: support for a role of triggering in causation. *Circulation*.

[18] Lampert R, Shusterman V, Burg MM (2005). Effects of psychologic stress on repolarization and relationship to autonomic and hemodynamic factors. *Journal of Cardiovascular Electrophysiology*.

[19] Kop WJ, Krantz DS, Nearing BD (2004). Effects of acute mental stress and exercise on T-wave alternans in patients with implantable cardioverter defibrillators and controls. *Circulation*.

[20] Lampert R, Shusterman V, Burg M (2009). Anger-induced T-wave alternans predicts future ventricular arrhythmias in patients with implantable cardioverter-defibrillators. *Journal of the American College of Cardiology*.

[21] Berger RD, Kasper EK, Baughman KL, Marban E, Calkins H, Tomaselli GF (1997). Beat-to-beat QT interval variability: novel evidence for repolarization lability in ischemic and nonischemic dilated cardiomyopathy. *Circulation*.

[22] Atiga WL, Calkins H, Lawrence JH, Tomaselli GF, Smith JM, Berger RD (1998). Beat-to-beat repolarization lability identifies patients at risk for sudden cardiac death. *Journal of Cardiovascular Electrophysiology*.

[23] Haigney MC, Zareba W, Gentlesk PJ (2004). QT interval variability and spontaneous ventricular tachycardia or fibrillation in the Multicenter Automatic Defibrillator Implantation Trial (MADUT) II patients. *Journal of the American College of Cardiology*.

[24] Piccirillo G, Magrì D, Matera S (2007). QT variability strongly predicts sudden cardiac death in asymptomatic subjects with mild or moderate left ventricular systolic dysfunction: a prospective study. *European Heart Journal*.

[25] Piccirillo G, Magrì D, Ogawa M (2009). Autonomic nervous system activity measured directly and QT interval variability in normal and pacing-induced tachycardia heart failure dogs. *Journal of the American College of Cardiology*.

[26] Ogawa M, Zhou S, Tan AY (2007). Left stellate ganglion and vagal nerve activity and cardiac arrhythmias in ambulatory dogs with pacing-induced congestive heart failure. *Journal of the American College of Cardiology*.

[27] Piccirillo G, Magrì D, Matera S, Marigliano V (2008). Emotions that afflict the heart: influence of the autonomic nervous system on temporal dispersion of myocardial repolarization. *Journal of Cardiovascular Electrophysiology*.

[28] Piccirillo G, Rossi P, Magrì D (2011). The QT variability index: a multidimensional approach to understanding cardiovascular disease. *Cardiology*.

[29] Task Force of the European Society of Cardiology and the North American Society of Pacing and Electrophysiology (1996). Heart rate variability. Standard of measurements, physiological interpretation and clinical use. *Circulation*.

[30] Piccirillo G, Nocco M, Moisè A (2003). Influence of vitamin C on baroreflex sensitivity in chronic heart failure. *Hypertension*.

[31] Piccirillo G, Cacciafesta M, Viola E (2001). Influence of aging on cardiac baroreflex sensitivity determined non-invasively by power spectral analysis. *Clinical Science*.

[32] Piccirillo G, Magrì D, Naso C (2004). Factors influencing heart rate variability power spectral analysis during controlled breathing in patients with chronic heart failure or hypertension and in healthy normotensive subjects. *Clinical Science*.

[33] Magrì D, Piccirillo G, Bucci E (2012). Increased temporal dispersion of myocardial repolarization in myotonic dystrophy type 1. Beyond the cardiac conduction system. *International Journal of Cardiology*.

[34] Magrì D, Sciomer S, Fedele F (2007). Increased QT variability in young asymptomatic patients with *β*-thalassemia major. *European Journal of Haematology*.

[35] Piccirillo G, Ogawa M, Song J (2009). Power spectral analysis of heart rate variability and autonomic nervous system activity measured directly in healthy dogs and dogs with tachycardia-induced heart failure. *Heart Rhythm*.

[36] Guzzetti S, La Rovere MT, Pinna GD (2005). Different spectral components of 24h heart rate variability are related to different modes of death in chronic heart failure. *European Heart Journal*.

[37] La Rovere MT, Pinna GD, Maestri R (2003). Short-term heart rate variability strongly predicts sudden cadiac death in chronic heart failure patients. *Circulation*.

[38] Piccirillo G, Magrì D, Di Carlo S (2006). Influence of cardiac-resynchronization therapy on heart rate and blood pressure variability: 1-year follow-up. *European Journal of Heart Failure*.

[39] Piccirillo G, Luparini RL, Celli V (2000). Effects of carvedilol on heart rate and blood pressure variability in subjects with chronic heart failure. *American Journal of Cardiology*.

[40] Piccirillo G, Quaglione R, Nocco M (2002). Effects of long-term beta-blocker (metoprolol or carvedilol) therapy on QT variability in subjects with chronic heart failure secondary to ischemic cardiomyopathy. *American Journal of Cardiology*.

[41] Piccirillo G, Magnanti M, Matera S (2006). Age and QT variability index during free breathing, controlled breathing and tilt in patients with chronic heart failure and healthy control subjects. *Translational Research*.

[42] Tsuji Y, Zicha S, Qi XY, Kodama I, Nattel S (2006). Potassium channel subunit remodeling in rabbits exposed to long-term bradycardia or tachycardia: discrete arrhythmogenic consequences related to differential delayed-rectifier changes. *Circulation*.

[43] Lampert R, Joska T, Burg MM, Batsford WP, McPherson CA, Jain D (2002). Emotional and physical precipitants of ventricular arrhythmia. *Circulation*.

[44] Kohn CS, Petrucci RJ, Baessler C, Soto DM, Movsowitz C (2000). The effect of psychological intervention on patients’ long-term adjustment to the ICD: a prospective study. *Pacing and Clinical Electrophysiology*.

[45] Pedersen SS, van den Broek KC, Sears SF (2007). Psychological intervention following implantation of an implantable defibrillator: a review and future recommendations. *Pacing and Clinical Electrophysiology*.

